# Supplementary material for: Specific Isolation of Clostridium botulinum Group I Cells by Phage Lysin Cell Wall Binding Domain with the Aid of S-Layer Disruption
Source: Int J Mol Sci. 2022 Jul 29;23(15):8391. doi: 10.3390/ijms23158391 (PMC9368847; doi:10.3390/ijms23158391)
Supplement: Supplementary file 1 [file ijms-23-08391-s001.zip › ijms-1763256-supplementary.pdf]

Supplementary Material

Table S1. Bacterial strains.

**Bacterial strain or plasmid**	**Relevant characteristics**	**Reference or source**[a]
Clostridium botulinum		
ATCC3502	Group I, produces type A botulinum neurotoxin (BoNT/A)	(54)
NCTC2916	Group I, produces BoNT/A, carries a silent gene for BoNT/B	(55)
62A	Group I, produces BoNT/A	(55)
ATCC19397	Group I, produces BoNT/A	(56)
133-4803	Group I, produces BoNT/B	(57)
213B	Group I, produces BoNT/B	(57)
F Langeland	Group I, produces BoNT/F	(55)
Eklund 2B	Group II, produces BoNT/B	(55)
CB11/1-1	Group II, produces BoNT/E	(58)
K126	Group II, produces BoNT/E	(58)
Eklund 202F	Group II, produces BoNT/F	(55)
BKT2873	Group III, produces BoNT/CD	(59)
**Other species**		
Clostridium sporogenes NINF45	Nontoxigenic, closely related to *C. botulinum* Group I	(31)
Clostridium baratii CCUG24033	Nontoxigenic, Also called *C. botulinum* Group V, type strain	(31)
Clostridium butyricum BL86/13	Also called C. botulinum Group VI, produces BoNT/E	DFHEH/QI
Clostridium perfringens ATCC13124	Gas gangrene isolate, produces α-toxin	(60)
Bacillus cereus ATCC14579	*B. cereus* type strain	ATCC
Bacillus subtilis 1012M15	Derivative strain from *B. subtilis* 168	(12)
Listeria monocytogenes EGD-e	Serotype 1/2a, causes listeriosis	(61)
Staphylococcus aureus ATCC12600	*S. aureus* type strain	(62)
Escherichia coli 5 alpha	Derivative strain of *E. coli* DH5α	NEB
Escherichia coli Rosetta 2(DE3) pLysS	F^{-} *ompT* *hsdS*$_B$(r_B^{-} m_B^{-}) *gal dcm* (DE3) pLysSpRARE2[3] (Cam^R)	Merck

[a] DFHEH, Department of Food Hygiene and Environmental Health, Faculty of Veterinary Medicine, University of Helsinki, Finland. QI, Quadram Institute, United Kingdom. ATCC, American Type Culture Collection.

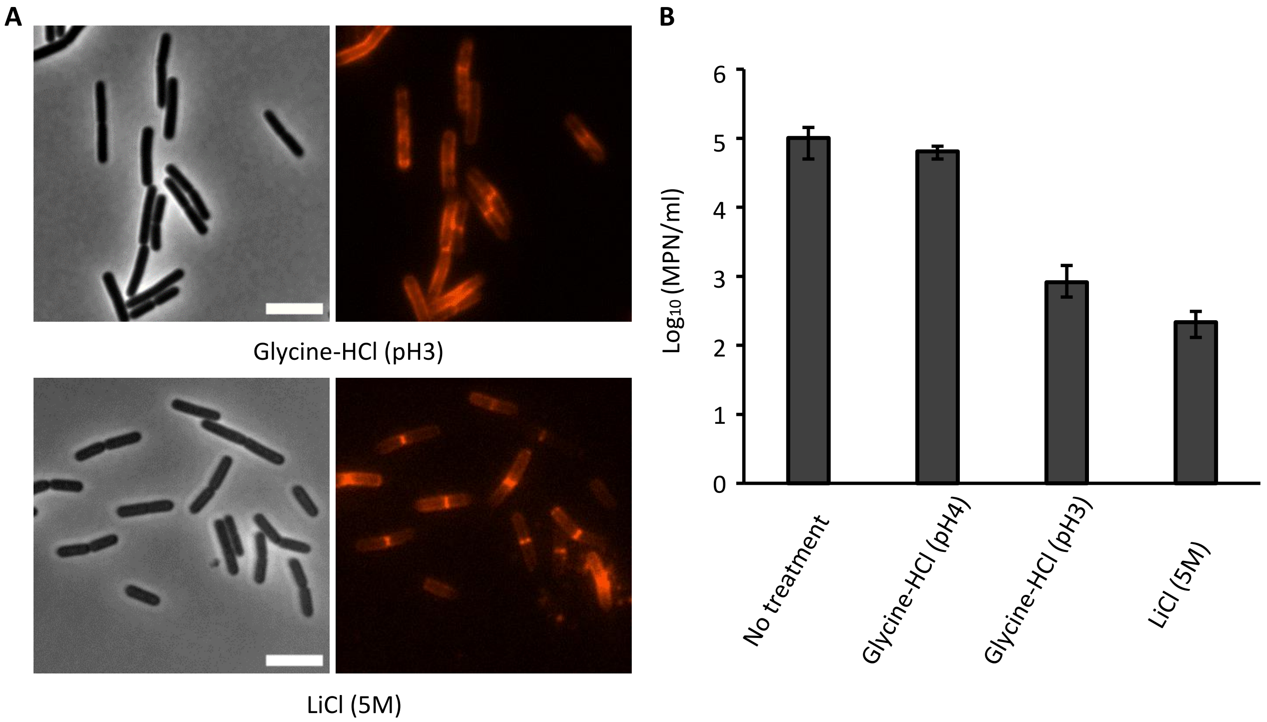

Figure S1. Treatment of *C. botulinum* ATCC3502 vegetative cells with glycine-HCl (pH3) and LiCl. (**A**) Representative fluorescence images showing the binding of mCherry-CBD to *C. botulinum* ATCC3502 cells after treated with 0.2M glycine-HCl (pH3) and 5M LiCl. Bars, 5 µm. (**B**) Most probable number (MPN) enumeration of equal fractions of *C. botulinum* ATCC3502 vegetative cells that were either no treatment or treated with 0.2 M glycine-HCl (pH4), glycine-HCl (pH3) or 5M LiCl for 1 min. Results are presented as means of three replicates ± standard deviations.

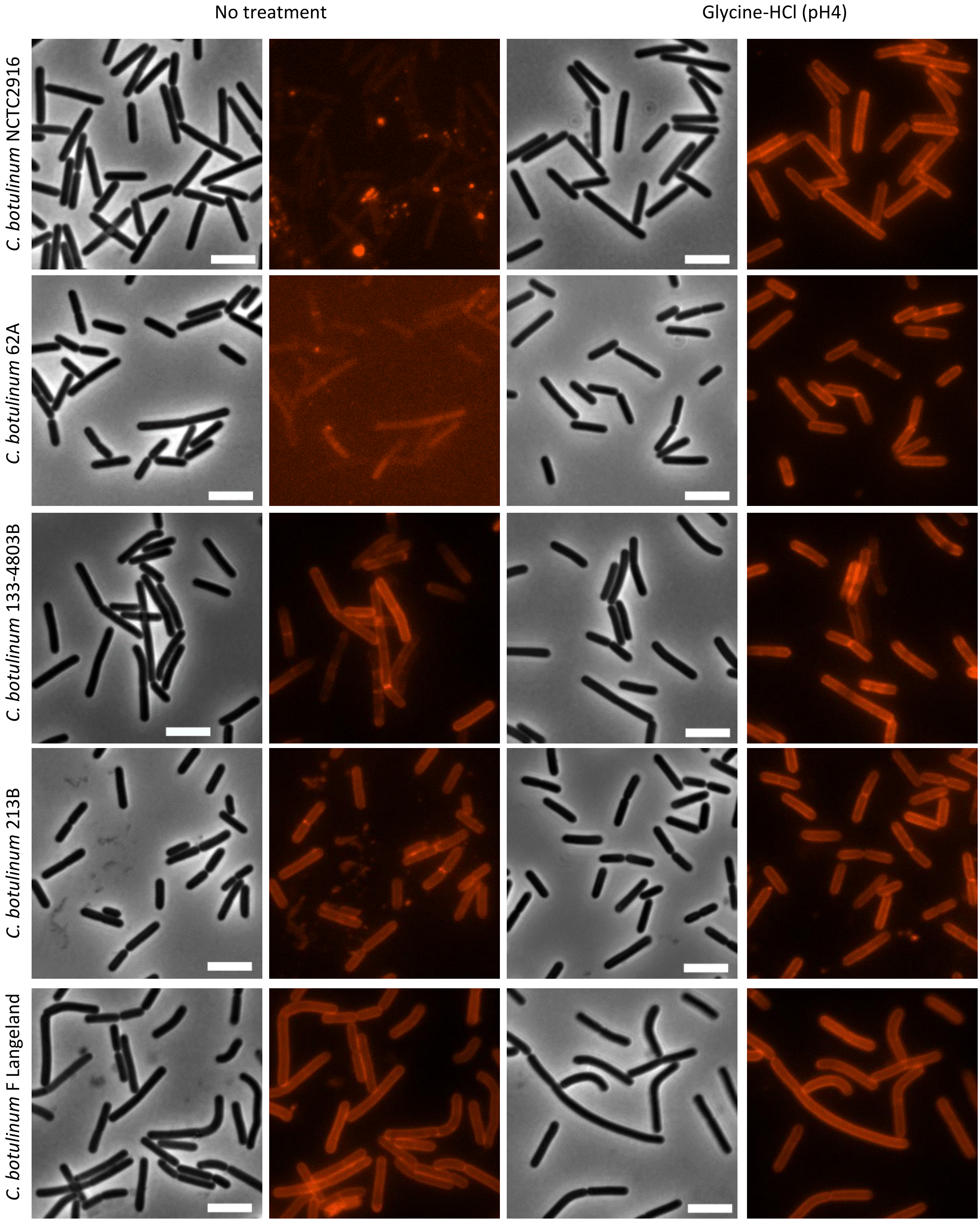
No treatment
Glycine-HCl (pH4)
C. botulinum NCTC2916
C. botulinum 62A
C. botulinum 133-4803B
C. botulinum 213B
C. botulinum F Langeland

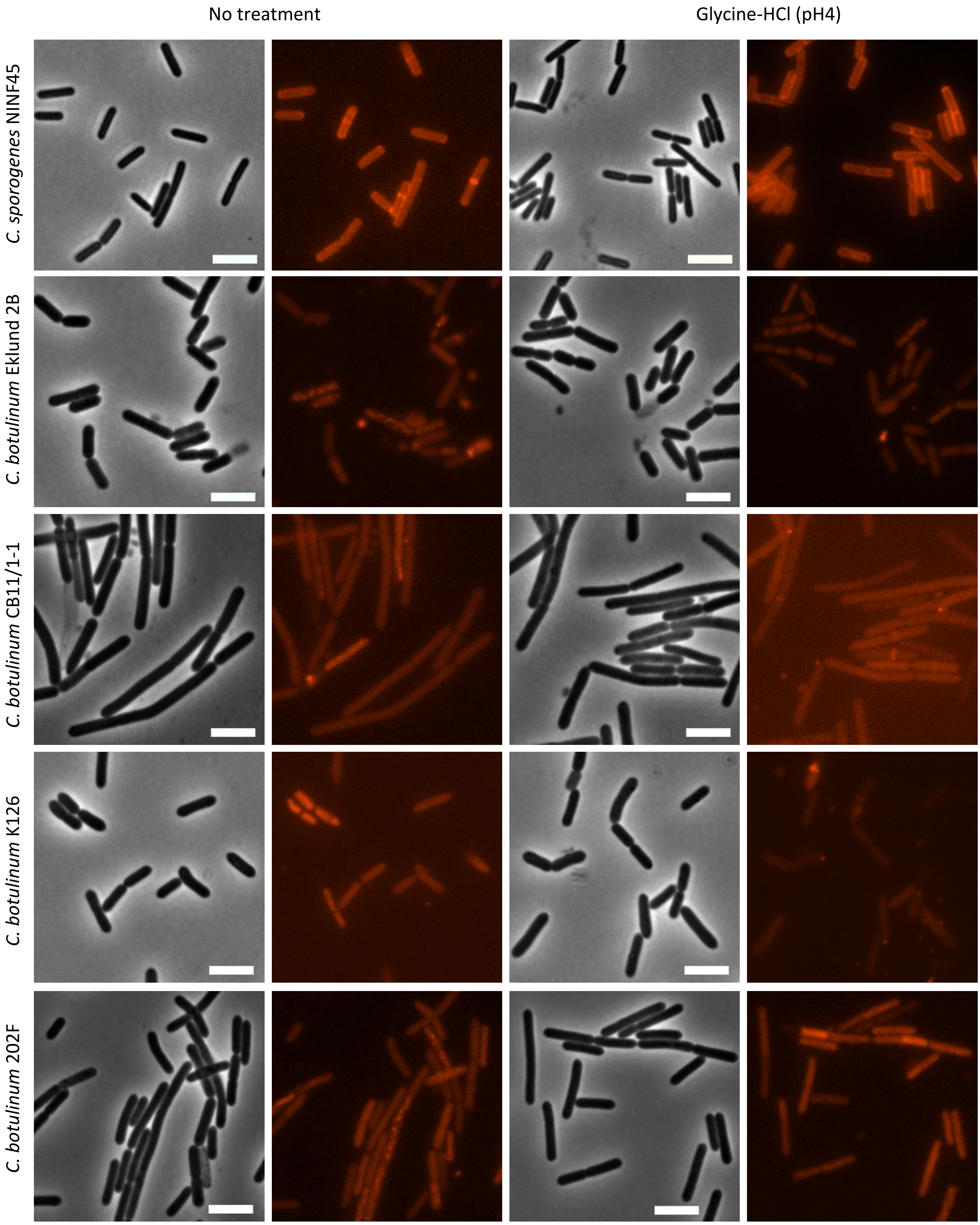

Figure S2 (continued)

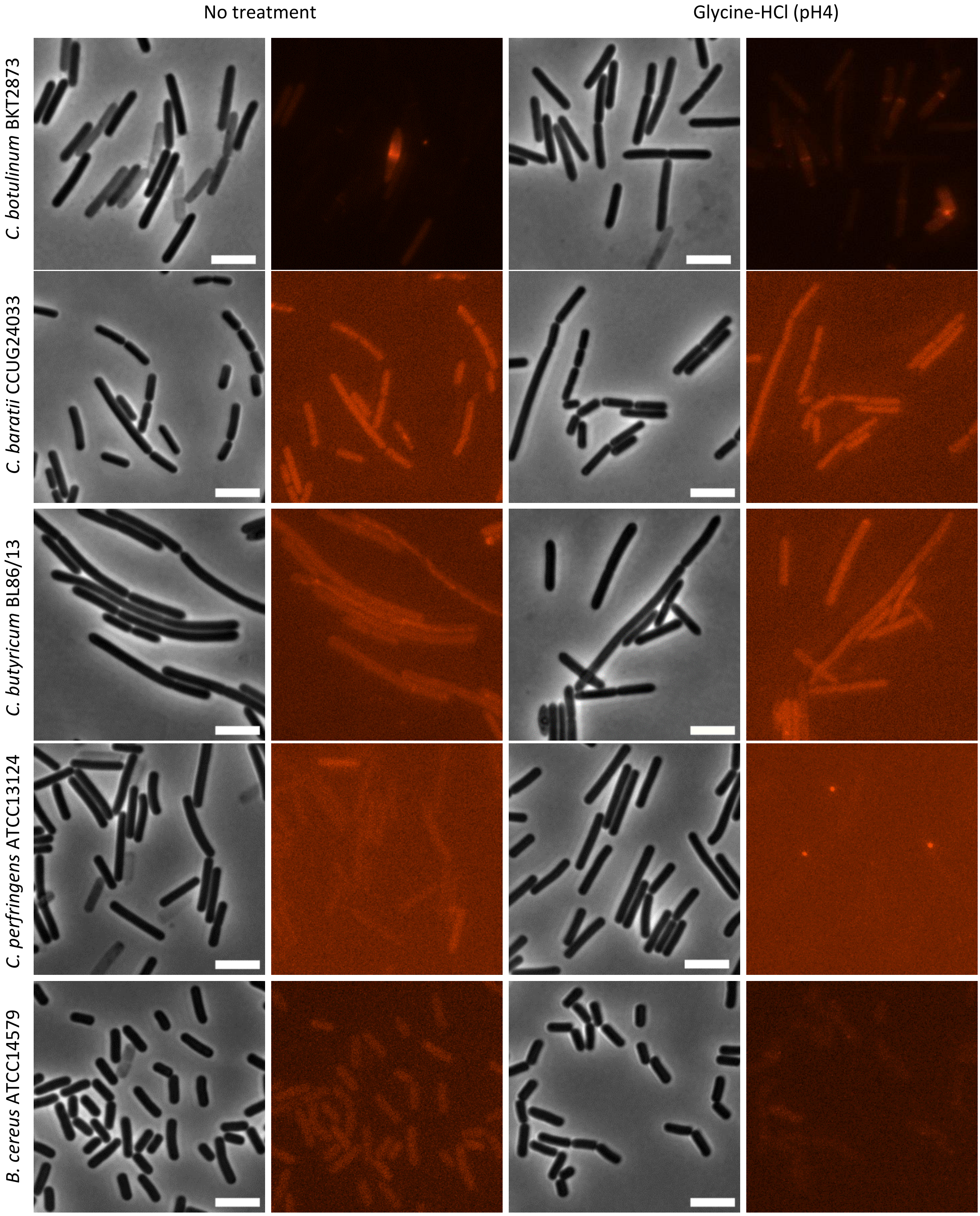

Figure S2 (continued)

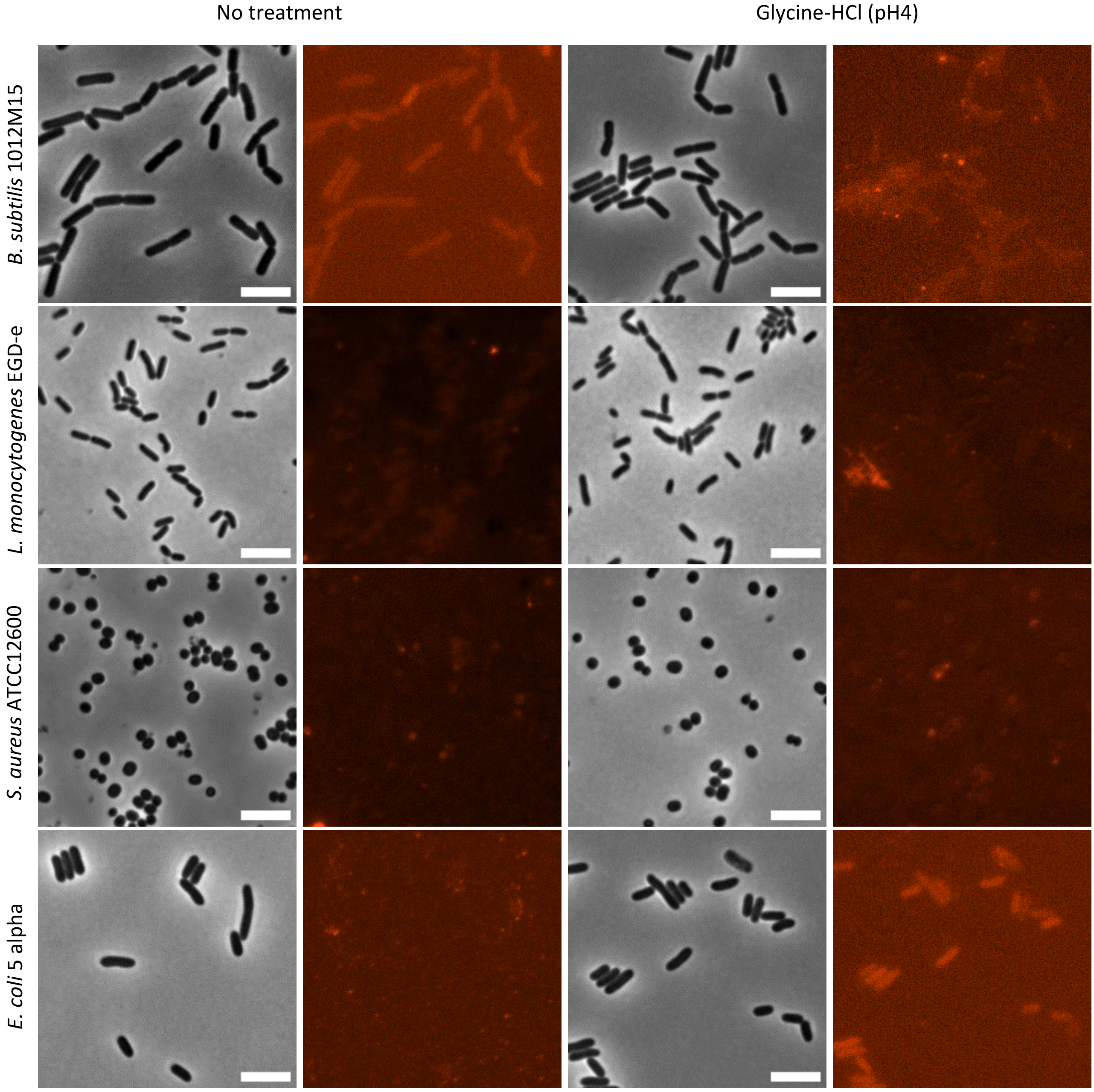

Figure S2. Representative fluorescence images of bacterial vegetative cells after incubation with mCherry-CBD. Before that, cells were either no treatment or treated with 0.2 M Glycine-HCl (pH 4) for 1 min. Bars, 5 µm.

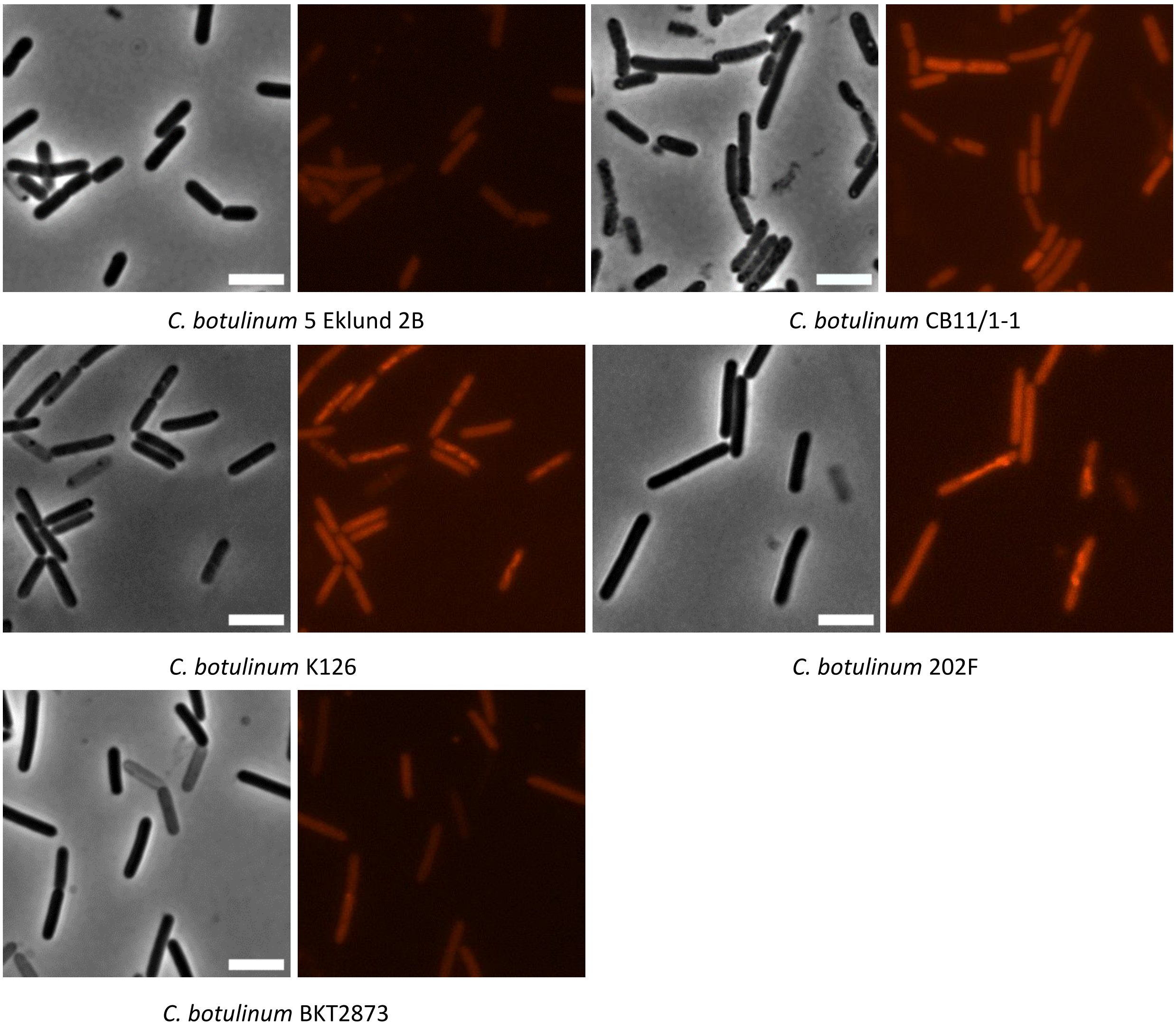

Figure S3. Representative images of *C. botulinum* Group II and Group III vegetative cells showing autofluorescence when viewed with a Texas Red filter.

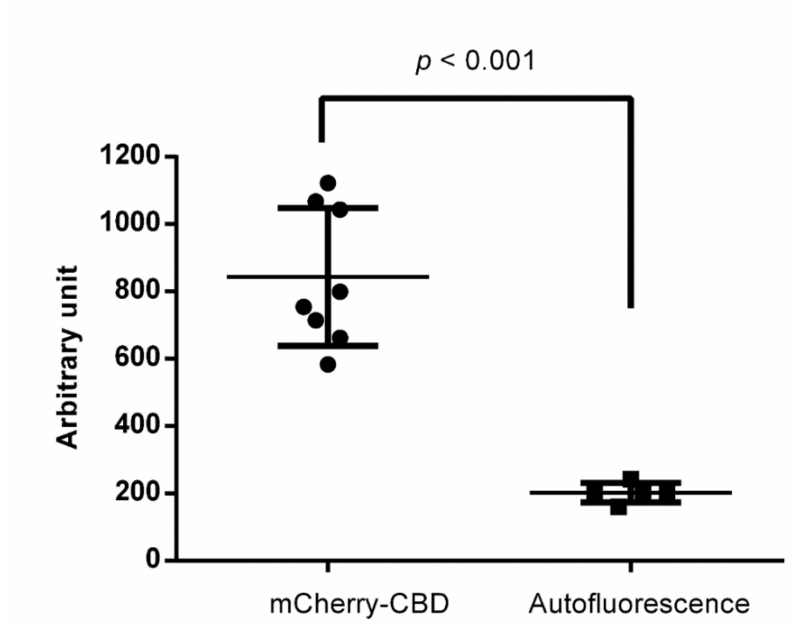

Figure S4. Comparison of fluorescence intensity of mCherry-CBD-labeled cells and autofluorescent cells. Fluorescence intensity of mCherry-CBD was measured from seven *C. botulinum* Group I strains and *C. sporogenes* NINF45. Autofluorescence intensity was measured from five *C. botulinum* Group II and Group III strains. Student's *t*-test was used for statistical analysis.

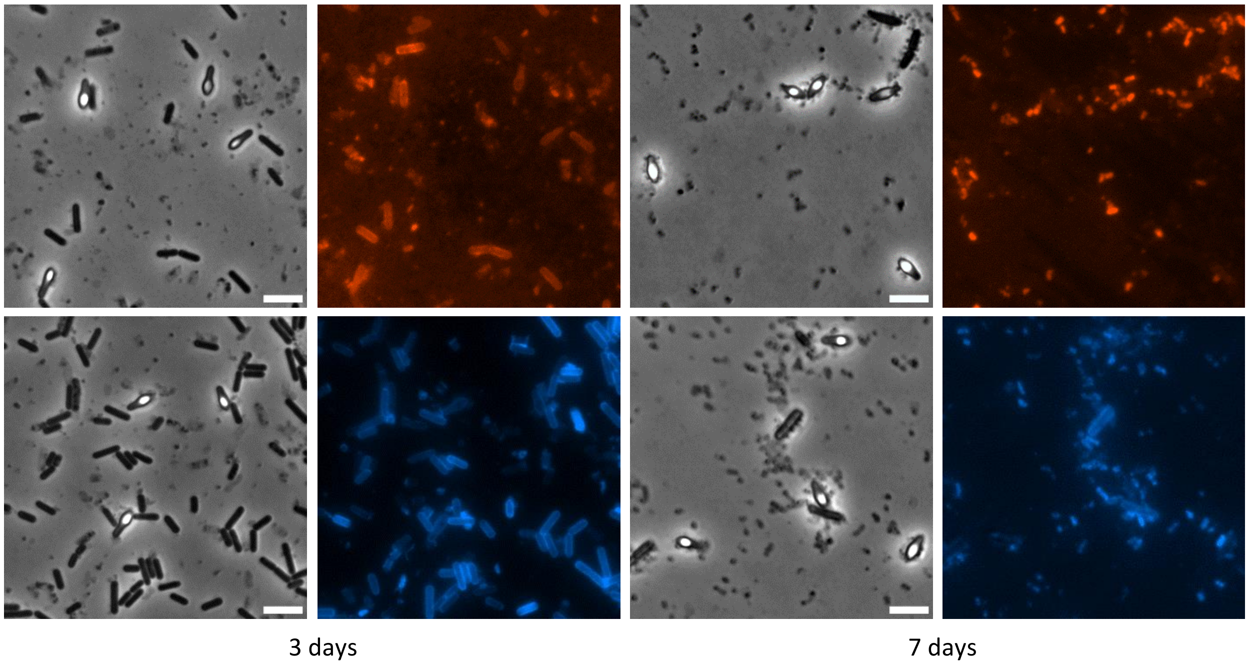

Figure S5. Representative fluorescence images of *C. botulinum* ATCC19397 cells cultured for 3 and 7 days after incubation with mCherry-CBD and mTagBFP-CBD. Bars, 5 µm.

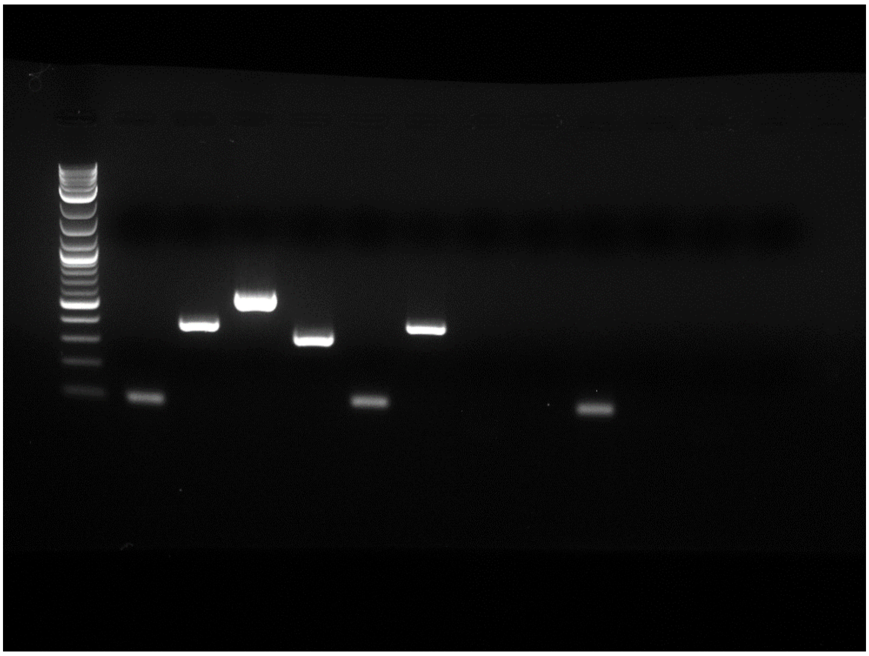

Figure S6. Original gel image used to prepare Figure 3C.

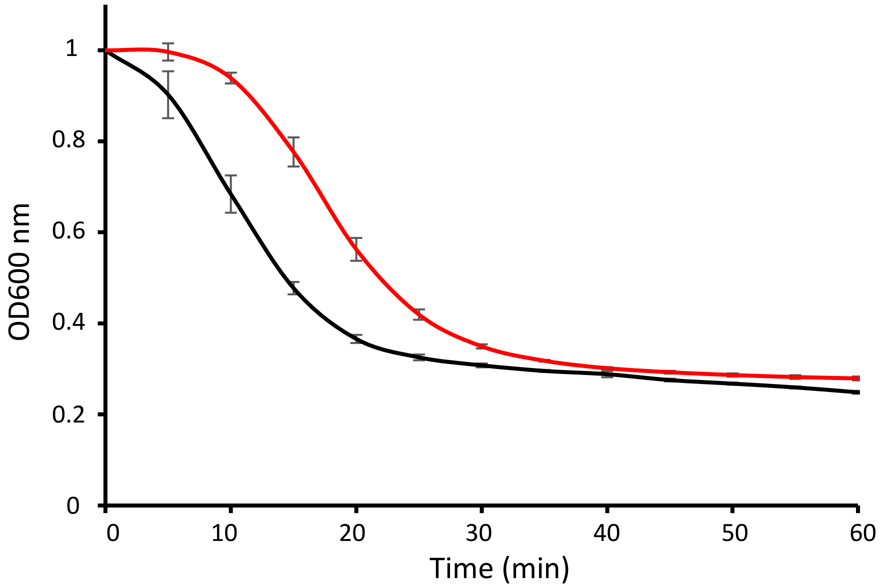

Figure S7. Turbidity reduction assay by measuring OD_{600} of vegetative cells of *C. botulinum* Group I ATCC3502 (red line) and ATCC19397 (black line) over 60 min after addition of 5 µM of lysin CBO1751. All OD_{600} values were normalized to an initial OD_{600} of 1. Data are presented as means of three replicates ± standard deviations.
